# Innovative Recruitment Strategies to Increase Diversity of Participation in Parkinson’s Disease Research: The Fox Insight Cohort Experience

**DOI:** 10.3233/JPD-191901

**Published:** 2020-04-03

**Authors:** Roseanne D. Dobkin, Ninad Amondikar, Catherine Kopil, Chelsea Caspell-Garcia, Ethan Brown, Lana M. Chahine, Connie Marras, Nabila Dahodwala, Sneha Mantri, David G. Standaert, Marissa Dean, Ira Shoulson, Kenneth Marek, Andrea Katz, Monica Korell, Lindsey Riley, Caroline M. Tanner, on behalf of the Fox Insight Study

**Affiliations:** a Rutgers, The State University of New Jersey, Piscataway, NJ, USA; b The Michael J. Fox Foundation for Parkinson’s Research, New York, NY, USA; c University of Iowa, Iowa City, IA, USA; d University of California, & San Francisco Veterans Affairs Medical Care Plan, San Francisco, San Francisco, CA, USA; e University of Pittsburgh, Pittsburgh, PA, USA; f Toronto Western Hospital, Toronto, Canada; g University of Pennsylvania, Philadelphia PA, USA; h Duke University, Durham, NC, USA; i University of Alabama at Birmingham, Birmingham, AL, USA; j University of Rochester, Rochester, NY, USA; k Institute for Neurodegenerative Disorders, New Haven, CT, USA

**Keywords:** Parkinson’s disease, diversity, recruitment, clinical trial

## Abstract

**Background:**

Clinical research in Parkinson’s disease (PD) faces practical and ethical challenges due to two interrelated problems: participant under-recruitment and lack of diversity. Fox Insight (FI) is a web-based longitudinal study collecting patient-reported outcomes and genetic data worldwide to inform therapeutic studies. FI’s online platform provides an opportunity to evaluate online strategies for recruiting large, diverse research cohorts.

**Objective:**

This project aimed to determine 1) whether FI’s digital marketing was associated with increased enrollment overall and from under-represented patient groups, compared to traditional recruitment methods; 2) the clinical and demographic characteristics of samples recruited online, and 3) the cost of this online recruitment.

**Method:**

FI recruitment during a 6-week baseline period without digital promotion was compared to recruitment during several periods of digital outreach. Separate online recruiting intervals included general online study promotion and unique Facebook and Google ad campaigns targeting under-represented subgroups: early PD, late/advanced PD, and residents of underrepresented/rural geographic areas.

**Results:**

Early PD, late PD, and geotargeting campaigns enrolled more individuals in their respective cohorts compared to baseline. All online campaigns also yielded greater total FI enrollment, attracting more participants who were non-White, Hispanic, older, female, and had lower educational attainment and income, and more medical comorbidities. Cost per new participant ranged from $21 (Facebook) to $108 (Google).

**Conclusion:**

Digital marketing may allow researchers to increase, accelerate, and diversify recruitment for PD clinical studies, by tailoring digital ads to target PD cohort characteristics.

## INTRODUCTION

Participant under-recruitment, and the related problem of homogenous research cohorts, pose practical and ethical challenges to advancing clinical research in Parkinson’s disease (PD) [1, 2]. While these problems hinder clinical research at large—causing the termination or substantial under-enrollment of 19% of registered trials [3]—PD researchers confront especially severe recruitment challenges. Estimates suggest that insufficient enrollment prevents completion of 30% of PD clinical trials and delays 85% of those eventually completed [4]. In addition, research that is underpowered (due to small sample size) is at greater risk of failing to detect true therapeutic benefits, resulting in missed opportunities to develop potentially life-changing interventions [5].

Compounding these enrollment difficulties, PD study samples typically reflect the characteristics of patients treated in select movement disorder specialty centers linked to research development (i.e., predominantly white men of European ancestry and higher socioeconomic status [6, 7]). To the extent that these samples reflect the influence of unequal access to specialty PD care, rather than the true diversity of the PD population, they limit the generalizability of research findings and their value for understanding the scope of PD heterogeneity [8–12]. Given the marked variability in PD symptom presentation and progression, future studies will require more precisely defined PD cohorts, in pursuit of the personalized medicine needed to advance clinical care and the race for the cure [13]. Under such targeted inclusion criteria, achieving necessary sample sizes becomes even more difficult, requiring novel recruitment strategies with broad reach. As such, initiatives to improve patient-centered recruitment and retention remain a priority in PD clinical research [14], where online tools are expanding and accelerating these efforts [15].

Launched by the Michael J. Fox Foundation for Parkinson’s Research in 2017, Fox Insight (FI) is a web-based longitudinal study which collects patient-reported outcomes (PROs) and genetic data from participants worldwide, including those with manifest and prodromal parkinsonism, and their caregivers, with the goal of informing therapeutic studies [16]. With a recruitment goal of over 125,000 volunteers, FI stands to dramatically improve not only the accessibility of clinical studies for people with PD (PWP), but also the statistical power (sample size), and diversity of PRO data over the current state of research. Importantly, the FI online data collection platform affords a unique opportunity to evaluate innovative recruitment techniques aimed at attracting large, diverse research cohorts.

Towards that end, this study evaluated the use of digital media to both fast-track and diversify study enrollment by targeting PWP cohorts who are traditionally under-represented in clinical research. The first of our three aims involved examining whether PD-specific digital marketing campaigns increased *targeted* and *overall* FI enrollment compared to baseline periods lacking paid promotion. The *targeted* campaigns recruited three subgroups: individuals with early stage PD, those with late stage/more advanced PD, and U.S. rural-dwelling PWP; general online recruitment drew from the broader PD community. Second, we aimed to clinically and demographically compare digitally recruited samples to those enrolled during baseline “recruitment as usual”; and third, to compare the reach and cost of different digital recruitment methods. We hypothesized that digital marketing would accelerate study recruitment and effectively target under-represented cohorts to increase their participation in the FI longitudinal cohort.

## METHODS

### Participants

Individuals aged 18 and over and English-literate were eligible to enroll in FI. Participants were informed that the study aimed to improve understanding of PD, such as its impact on health and quality of life over time and possible connections between family neurological history, general health, environmental risk factors, and PD. Participants affirmed an online statement of informed consent prior to the initiation of any study procedures. FI maintains the full approval of the New England IRB. Since 7/30/14 (first registration in pilot phase), approximately 42,000 individuals have enrolled.

Participants who self-reported a PD diagnosis from a healthcare provider were identified as participants with PD (PWP). In a subset of individuals, the PD diagnosis was confirmed via telehealth consultation with a movement disorders specialist. The rate of diagnostic agreement (between self-report and expert consultation) was high (kappa = 0.85; 95% CI–0.76–0.94) (Schneider RB, Myers TL, Daeschler M, Tarolli C, Adams J, Barbano R, Riley L, Amondikar N, Auinger P, Diaz M, Dorsey ER, Marras C, Tanner C, Validation of Fox Insight cohort via virtual research visits). PWP were the largest cohort in this observational design and the target audience for the digital marketing campaigns described below, though FI additionally enrolls healthy controls, caregivers and those who may be at increased PD risk (e.g., REM Behavior Disorder; Hyposmia; known PD genetic mutation), compared to the general population.

### Measures

Patient-reported outcomes (PROs) are becoming increasingly important to research, therapeutic development and healthcare delivery. As such, FI captures detailed demographics; medical and psychiatric history; family neurological history; and participant responses to validated questionnaires on physical health, mental health, and health-related quality of life. Self-report measures include the Non-Motor Symptom Questionnaire (NMS Quest), MDS-Unified Parkinson’s Disease Rating Scale (UPDRS) Part II, Geriatric Depression Scale (GDS), Parkinson’s Disease Quality of Life Inventory (PDQ-8), Penn Parkinson’s Daily Activities Questionnaire (PDAQ-15), and a brief REM Behavior Disorder (RBD) symptom assessment [17–22]. Once enrolled, participants are asked to provide updated responses every 90 days for several years. To enhance compliance, participants are automatically prompted, twice via email (the day the study visit opens and 3 days before it will close), to complete their next study visit, during the appropriate time interval.

Prior to the official launch of the study, the FI platform was extensively pilot tested by PWP, caregivers, and healthy controls, with appropriate modifications made to optimize the user experience based on participant feedback regarding site navigation and visual appeal. All FI data is publicly available to the research community via the “FOX DEN” (Fox Insight Data Exploration Network): https://foxden.michaeljfox.org/.

Several additional metrics captured data describing the visibility, reach, and cost of each targeted digital campaign (Groups 2, 3, and 5 as described further below). These included the numbers of individuals who 1) viewed the ad, 2) clicked on the ad, and 3) enrolled in FI via the ad’s link, as well as 4) cost per recruited subject. “Recruitment” was defined as completing required registration information and providing informed consent.

### Procedure

#### Overview

A baseline control (pre-intervention period) was defined as six weeks with no paid digital promotion (Group 1; baseline; “Recruitment as usual”) and was compared to unique online ad campaigns targeting late-stage or more advanced PD (Group 2; “Late PD”), early-stage PD (Group 3; “Early PD”), and U.S. rural-dwelling PWP (Group 5; “Geotargeting”), as well as to a more general online study promotion (Group 4; “Broad online recruitment”). Facebook was used to recruit individuals for the late PD (Group 2) and geotargeting (Group 5) campaigns, respectively, while Google Search Engine Marketing (SEM) was used to attract new FI participants with early PD (Group 3). The rationale for selecting these digital platforms for each particular campaign is outlined below.

Late/more advanced PD was defined as disease duration greater than 10 years or an NMS-Quest score greater than 13 or an MDS-UPDRS Part II score greater than 25 [23, 24]. Early-stage PD was defined as disease duration less than 3 years. Regarding Group 5, a list of zip codes from non-urban areas (per the U.S. Census Bureau [25]), in select Western and Midwestern U.S. states were targeted, as the overwhelming majority of FI participants were then concentrated on the U.S. east and west coasts.

**Fig.1:**
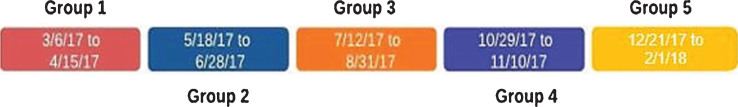
Timeline of Each Unique Recruitment Campaign. Note: Group definitions. Group 1: Baseline recruitment (control group). Group 2: Facebook late-stage. Group 3: Google early-stage. Group 4: Broad online. Group 5: Facebook Geo-targeting.

All digital advertisements were IRB-approved and displayed hyperlinks that directed individuals to the FI website (www.foxinsight.org). Campaign timelines are shown in Fig. 1. All promotional campaigns were run serially and sequentially as described below; no two campaigns ran simultaneously.

#### Baseline “Recruitment as Usual”

*Group 1: Baseline “Recruitment as usual”.* This group incorporated 1) educational content and study information on the MJFF website with no additional effort to drive PWP towards this information; 2) a study listing on Fox Trial Finder (https://foxtrialfinder.michaeljfox.org), a website where PWP can locate information about active PD studies and be directed towards those for which they may qualify; 3) live promotion at MJFF-sponsored community events; and 4) routine “on the ground” distribution of study flyers in the PD community (support groups, neurology clinics).

#### Digital Marketing Campaigns

*Group 2: Facebook Campaign for “Late PD”.* Facebook was chosen as the digital platform for the late PD campaign for several reasons. First, research suggests that individuals who have been living with a chronic medical condition, such as PD, for a longer period may be more open about their diagnosis and more likely to post their interests in and support for disease-related groups on social media [26, 27]. Second, the prevalence of internet use among older adults in general, and Facebook use in particular, has climbed substantially in recent years [28–30], making online advertising a feasible mechanism for recruitment. Lastly, Facebook offers researchers the ability to advertise to narrowly defined segments of their user community based on individual users’ “interests” indicated in their personal profiles (i.e., interest targeting).

This six-week pilot campaign leveraged Facebook’s algorithm to target users age 60 and over who indicated interests on the site in both “Parkinson’s disease awareness” and at least one additional PD-related subject area (e.g., clinical trials, PD symptoms and PD organizations; see Supplementary Figure 1). The Facebook advertising campaign was divided into three two-week phases to test and optimize various aspects of the promotion.

The first phase tested two images, pairing each with two unique headlines (four total combinations; Supplementary Figure 2, Panels 1–4). The best-performing ad image and headline combination (Supplementary Figure 2, Panel 3) was then used to test interest targeting based on two Facebook interests: *PD symptoms* and *PD research*. In the *PD symptoms ad* group, the interest targeting was set up to match the “Parkinson’s disease awareness” interest and one of the following interests related to late-stage/more advanced PD: cognitive deficit, dyskinesia, insomnia, gait, dementia, restless legs syndrome, movement disorder, or any of 14 additional similar topics. The *PD research ad* group was likewise set up to match “Parkinson’s disease awareness” and one of the following interests: Parkinson’s disease clinical research, clinical trial, clinical research, medical cannabis, clinicaltrials.gov, or phases of clinical research.

Phase 3 ran the same optimized ad sets from Phase 2 (Supplementary Figure 2, Panel 3) using the broad parameter of people within the U.S. over 60 years of age. The goal of this broad targeting was to expand the ad audience to include Facebook users who did not fit the initial parameters but might still fit study recruitment criteria.

*Group 3: Google Search Engine Marketing Campaign for “Early PD”.* Google Search Engine Marketing (SEM) was chosen over Facebook as the digital platform for individuals with early PD based on prior findings that newly diagnosed PWP may be more reluctant to share their diagnosis publicly on social media profiles [31, 32], but may be highly likely to research their new diagnoses online [26, 33]. This campaign ran for four weeks and displayed targeted advertisements with Google Search results.

Advertisements were presented to select individuals in response to “keywords” they used when conducting internet searches. Possible search terms that newly diagnosed individuals might use when learning about PD online (e.g., Parkinson’s early signs, Parkinson’s stages, Parkinson’s symptoms, etc.) were piloted tested to determine how frequently the phrases were included in searches, based on Google’s own database of search queries (http://www.trends.google.com). The most frequently searched PD-related terms were selected for this campaign. Thirty-four search keywords (Supplementary Figure 3) were ultimately included, such as Parkinson’s prognosis, Parkinson’s causes, movement disorder specialist, Parkinson’s symptoms, and levodopa.

Search keywords and advertising budget were finalized with Google, prior to the initiation of the recruitment campaign. A sample ad may be found in Supplementary Figure 4.

*Group 4: Broad Online Recruitment Campaign for all PWP.* There was a two-week period (late October–early November 2017; see Fig. 1) when a broad range of recruitment tactics were employed, coinciding with the official media launch of the study. This period included the public announcement of FI, with Michael J. Fox appearing on CBS Sunday Morning to discuss the study, a subsequent press release [34], and initial email notification to the MJFF community of around 380,000 patient, family and supporter subscribers.

In addition, paid Facebook ads were deployed to target 1) FI website visitors who did not enroll, 2) those who matched Facebook interest targeting (similar to Group 2) but never visited the FI website, and 3) people with similar Facebook activity to those who enrolled in FI (lookalike targeting). Non-paid ads were also deployed through Facebook, Instagram, and Twitter on Michael J. Fox Foundation social media accounts. Lessons learned by conducting the Facebook and Google campaigns described above (Groups 2-3) were used to optimize advertising strategies in this broad online initiative.

*Group 5: Facebook Campaign for Geotargeting.* Facebook was selected for the geotargeting campaign for its ability to present advertisements selectively to individuals in pre-determined geographic areas. This campaign was conducted to attract new FI participants from non-urban areas [25] in the Midwestern and Western U.S. (Supplementary Figure 5) with few existing FI participants, despite the campaigns described above (Groups 1–4). Non-urban areas in selected underrepresented states were targeted, as these regions traditionally have fewer clinical sites and opportunities for participation in research.

The first of this campaign’s two phases ran for two weeks and included 6,749 zip codes across 13 states. Phase 2 ran for one week and focused on the four top-performing states, in which Phase 1 advertising yielded the most new participants for the lowest cost. Phase 2 expanded the geographic area from specific zip codes to statewide targets (Kansas, Nebraska, North Dakota, and Oklahoma) to enlarge the audience of potential FI participants. Phase 1 data suggested that limiting Facebook advertising to specific zip codes was associated with suboptimal ad visibility.

Within these under-represented areas, Phases 1 and 2 inclusion criteria for interest targeting were the same: people age 45 and over, and online interest in PD philanthropic organizations. The minimum age was lowered from 60 to 45 to ensure sufficient ad visibility, given that the targeted areas were less populated. The following audience segments were excluded in both phases: MJFF website visitors, indicating an interest in MJFF, and previous FI registrations.

### Overview of statistical methods

Statistical analyses were performed using SAS 9.4 (SAS Institute Inc., Cary, NC) on a data extract from September 19, 2018. To address study aim 1, Poisson regression was used to test whether each digital enrollment campaign increased recruitment of the target cohort, as well as overall recruitment, compared to “recruitment as usual” during the baseline period. In order to control for the length of the online recruitment campaigns (two vs. four vs. six weeks), we explored the average number of new participants enrolled per 10-day period during each campaign. To address study aim 2, simple linear models, Chi-square, or Fisher’s exact tests were used to compare key demographic and clinical variables (e.g., gender, race, ethnicity, medical comorbidities, prior clinical trial participation) between individuals recruited via digital channels vs. “recruitment as usual” (baseline). For aims 1 and 2, if the overall group comparison *p*-value was < 0.05, then all pairwise group comparisons were performed. *Post hoc* tests with *p*-values < 0.05 were considered significant given the exploratory nature of the study.

Descriptive statistics regarding the number of times an ad was viewed, clicked, and directly linked to study enrollment, as well as cost per each new enrolled participant, are reported for study aim 3 (Groups 2, 3, and 5). Cost per recruited participant was calculated by dividing the number of new participants recruited in response to a particular campaign by its overall cost.

Data collected during the initial visit (Study Visit 1) was used to inform the analyses below. Participants had a 90-day window to complete each study visit.

## RESULTS

A total of 7,877 new participants enrolled in FI across the five campaigns described above. Importantly, across all five recruitment groups, the majority (52% [baseline] to 68% [early PD]) reported being diagnosed with PD by a non-specialist provider, including primary care physicians and general neurologists.

### Targeted and total FI recruitment

Early PD, late PD, and geotargeting campaigns successfully enrolled more individuals in their respective participant cohorts compared to baseline (early: 134 vs. 38, *p* < 0.0001; late: 190 vs. 35, *p* < 0.0001; geotargeting: 65 vs.4, *p* < 0.0001). Results remained significant when controlling for the small differences in campaign length (Table 1A). For example, per 10-day period, the geotargeting campaign enrolled approximately 30 new registrants from under-represented zip codes, compared to 1 comparable new participant during baseline. All online campaigns (Groups 2–5) were also associated with greater total FI enrollment, compared to baseline (Table 1B).

**Table 1A:** Targeted Recruitment Per Group

Targeted Population	Baseline Recruitment	Targeted Recruitment	*p*-value
**Late Stage PD**	**Group 1**	**Group 2**	<0.0001
N Recruited	35	190
Recruited per 10 days	8.54	45.24
**Early Stage PD**	**Group 1**	**Group 3**	<0.0001
N Recruited	38	134
Recruited per 10 days	9.27	26.27
**Geo-Targeted Zip Code**	**Group 1**	**Group 5**	<0.0001
N Recruited	4	65
Recruited per 10 days	0.98	29.55
**PD Subjects**	**Group 1**	**Group 4**	<0.0001
N Recruited	88	4689
Recruited per 10 days	21.46	3606.92

**Table 1B:** Total Recruitment Per Group

	Total Recruited	Recruited per 10 days	*p*-value (vs. Group 1)
**Group 1 (Baseline)**	120	29.27	–
**Group 2 (Late Stage)**	1120	266.67	<0.0001
**Group 3 (Early PD)**	356	69.80	<0.0001
**Group 4 (Global Paid Online)**	5919	4553.08	<0.0001
**Group 5 (Geotargeting)**	362	164.55	<0.0001

Late Stage PD is defined as 10 or more years between diagnosis and registration OR MDS-UPDRS II≥25 OR NMS Quest ≥13. Early PD is defined as <3 years between diagnosis and registration. Note: *p*-values from Poisson regression.

### Clinical and demographic characteristics of targeted subgroups

In addition to the targeted variables (e.g., PD stage, zip code), there were significant differences in the clinical and demographic characteristics of the samples recruited via digital marketing channels vs. baseline “recruitment as usual.” For example, non-white recruitment was significantly higher in all digital enrollment campaigns compared to baseline (Group 1). Additionally, Hispanic recruitment was significantly higher in the broad online campaign (Group 4; *p* < 0.0001) and the late PD campaign (Group 2, *p* = 0.0154) compared to baseline. Digital marketing campaigns also attracted participants that were older, female, and had lower educational attainment and income, as well as those with more complicated medical histories, compared to baseline. Importantly, initiatives characterized by narrowly defined clinical criteria (such as early- and late-stage PD) recruited significantly more registrants who had never participated in prior PD research. See Tables 2A and 2B for a breakdown of clinical and demographic history by recruitment group.

**Table 2A:** Clinical and Demographic Characteristics by Recruitment Group

Variable	Group 1 (N = 88)	Group 2 (N = 516)	Group 3 (N = 231)	Group 4 (N = 4689)	Group 5 (N = 236)	*p*-value
**Age**						<0.0001
Mean (SD)	61.19 (12.1)	66.73 (8.7)^**^	63.97 (10.1)^**^	65.35 (9.4)^**^	64.37 (11.0)^**^
(Min, Max)	(23.0, 85.0)	(30.0, 88.0)	(30.0, 86.0)	(25.0, 94.0)	(29.0, 90.0)
**Sex**						0.0190
Female	48 (54.5%)	238 (46.1%)	115 (49.8%)	2042 (43.5%)^**^	96 (40.7%)^**^
Male	37 (42.0%)	264 (51.2%)	105 (45.5%)	2535 (54.1%)^**^	136 (57.6%)^**^
Missing	3 (3.4%)	14 (2.7%)	11 (4.8%)	112 (2.4%)	4 (1.7%)
**Education**						0.0021
Associate’s or less	26 (29.5%)	201 (39%)	105 (45.4%)^**^	1,665 (35.5%)	77 (32.6%)
Bachelor’s or higher	59 (67.1%)	296 (57.4%)	112 (48.4%)^**^	2,869 (61.2%)	149 (63.1%)
Prefer Not to Answer	0 (0.0%)	2 (0.4%)	2 (0.9%)	17 (0.4%)	3 (1.3%)
Missing	3 (3.4%)	17 (3.3%)	12 (5.2%)	138 (2.9%)	7 (3.0%)
**Income**						0.0003
Less than $50,000	19 (21.5%)^**^	170 (33%)^**^	70 (30.3%)	1,171 (24.9%)	61 (25.9%)
$50,000 or above	54 (61.4%)^**^	270 (52.4%)^**^	116 (50.1%)	2,820 (60.1%)	138 (58.5%)
Prefer Not to Answer	12 (13.6%)	58 (11.2%)	32 (13.9%)	558 (11.9%)	30 (12.7%)
Missing	3 (3.4%)	18 (3.5%)	13 (5.6%)	140 (3.0%)	7 (3.0%)
**Employment Status**						0.0274
Employed Full/Part time	29 (32.9%)	108 (20.9%)^**^	61 (26.4%)	1,253 (26.8%)	58 (24.6%)
Retired/Unemployed	53 (60.2%)	386 (74.8%)^**^	153 (66.3%)	3,266 (69.6%)	168 (71.2%)
Prefer Not to Answer	3 (3.4%)	4 (0.8%)	4 (1.7%)	30 (0.6%)	3 (1.3%)
**# of Medical Comorbidities**						<0.0001
Mean (SD)	4.18 (2.3)	4.72 (2.5)	4.98 (2.8)^**^	3.46 (2.1)^**^	3.42 (2.2)^**^
(Min, Max)	(0.0, 9.0)	(0.0, 14.0)	(0.0, 13.0)	(0.0, 15.0)	(0.0, 12.0)
Missing	9	80	41	247	18
**Prior Clinical Trial Participation**						<0.0001
Yes	29 (33.0%)	110 (21.3%)^**^	31 (13.4%)^**^	1240 (26.4%)	65 (27.5%)
No	56 (63.6%)	386 (74.8%)^**^	185 (80.1%)^**^	3296 (70.3%)	163 (69.1%)
Prefer Not to Answer	0 (0.0%)	2 (0.4%)	2 (0.9%)	11 (0.2%)	1 (0.4%)
Missing	3 (3.4%)	18 (3.5%)	13 (5.6%)	142 (3.0%)	7 (3.0%)

Note: Due to small numbers, categories were collapsed for *p*-values for Education (Associate’s or less vs. Bachelor’s or higher), Income (< $50,000 vs. $50,000 or above) and Employment (Employed full/part-time vs. Retired/Unemployed). Note: *p*-values from Chi-square or *t*-tests; ^**^indicates group difference of *p* < 0.05 (from baseline) in *post-hoc* contrasts. **Note: Group definitions.** Group 1: Baseline recruitment (control group). Group 2: Facebook late-stage. Group 3: Google early-stage. Group 4: Broad online. Group 5: Facebook Geo-targeting.

**Table 2B:** Minority Recruitment by Group

Group	Total Non-White Recruitment	Non-White Recruitment per 10 days	*p*-value (vs. Group 1)	Total Hispanic Recruitment	Hispanic Recruitment per 10 days	*p*-value (vs. Group 1)
**Group 1**	8	1.95	–	13	3.17	–
**Group 2**	40	9.52	<0.0001	29	6.90	0.0154
**Group 3**	27	5.29	0.0074	11	2.16	0.3459
**Group 4**	199	153.08	<0.0001	231	117.69	<0.0001
**Group 5**	17	7.73	0.0008	14	6.36	0.0721

Note: *p*-values from Poisson regression. **Note: Group definitions.** Group 1: Baseline recruitment (control group). Group 2: Facebook late-stage. Group 3: Google early-stage. Group 4: Broad online. Group 5: Facebook Geo-targeting.

*Late PD vs. early PD campaigns*. Clinical and demographic characteristics, as well as motor and non-motor symptom severity ratings, including disease duration and medication use, significantly differed between PWP recruited in the late vs. early PD campaigns. PWP recruited via the late stage PD Facebook campaign had PD for a longer period, were older, more likely to be retired, more highly educated, and more likely to be receiving symptomatic treatment for PD, compared to the those recruited via the early PD Google campaign (*p* < 0.05). Notable differences also emerged on questionnaire scores for individuals who met true late-stage vs. true early-stage PD criteria as defined by the protocol. Participants with late-stage PD reported more severe symptoms and functional impairment than early-stage participants on nearly all questionnaires; of exception, GDS scores revealed mild levels of depressive symptoms in both groups (Table 3).

**Table 3:** Motor and Non-Motor Questionnaire Scores in Late vs. Early Stage PD Subjects^**^

Variable^*^	Group 2 Late Stage (N = 190)	Group 3 Early Stage (N = 134)	*p*-value
**GDS Score**			0.5664
Mean (SD)	5.54 (3.7)	5.23 (4.0)
(Min, Max)	(0.0, 15.0)	(0.0, 15.0)
Missing	63	54
**Act out dreams while asleep**			<0.0001
Yes	84 (44.2%)	21 (15.7%)
No	69 (36.3%)	64 (47.8%)
Missing	37 (19.5%)	49 (36.6%)
**NMS Quest Score**			<0.0001
Mean (SD)	14.38 (4.0)	10.83 (5.7)
(Min, Max)	(0.0, 24.0)	(1.0, 24.0)
Missing	29	48
**MDS-UPDRS Part II Score**			<0.0001
Mean (SD)	16.45 (9.0)	10.15 (7.3)
(Min, Max)	(3.0, 45.0)	(1.0, 36.0)
Missing	42	49
**PDAQ-15 Score**			0.0007
Mean (SD)	15.15 (12.0)	9.74 (10.8)
(Min, Max)	(0.0, 57.0)	(0.0, 41.0)
Missing	39	49
**PDQ-8 Score**			<0.0001
Mean (SD)	28.16 (16.4)	19.57 (14.6)
(Min, Max)	(0.0, 75.0)	(0.0, 56.3)
Missing	24	42

^*^The Geriatric Depression Scale (GDS), Non-Motor Symptom Questionnaire (NMS Quest), Part II of the MDS-Unified Parkinson’s Disease Rating Scale (UPDRS), the Penn Parkinson’s Daily Activities Questionnaire (PDAQ-15), the Parkinson’s Disease Quality of Life Inventory (PDQ-8); acting out dreams while asleep is a symptom of REM behavior disorder (RBD). ^**^As defined by Study protocol. Note: Late Stage PD is defined as 10 or more years between diagnosis and registration OR MDS-UPDRS II≥25 OR NMS Quest≥13. Early Stage PD is defined as <3 years between diagnosis and registration. Note: *p*-values from Chi-square or *t*-tests.

### Visibility and cost of narrowly targeted campaigns

*Facebook Late Stage (Group 2):* The campaign to recruit late-stage PWP led to 1,268,323 individuals viewing the Facebook ad and 9,461 people clicking on the ad link to visit FI, at a total campaign cost of $23,945. In direct response to this campaign, 1,120 individuals enrolled in FI at a cost of $21.38 per new subject.

*Google Search Engine Marketing Early PD (Group 3):* The Google Ads were viewed by 426,457 people, resulting in 18,149 clicks on the ads, for a total campaign cost of $39,021. The campaign yielded 356 new FI participants, resulting in a cost of $107.79 per new subject.

*Facebook Geotargeting (Group 5):* This campaign reached 304,747 people, generating 3,364 link clicks, for a total campaign expenditure of $15,846. The 362 participants who enrolled had a cost of $43.77 per new subject.

## DISCUSSION

In order to truly advance Parkinson’s disease research and clinical care, innovative recruitment strategies are required to attract large numbers of research participants, as well as to accurately capture patient-reported outcomes (PROs) from diverse segments of the PD community. This paper describes three precision-targeted online advertising campaigns and one broad online campaign, all designed to supplement and extend the reach of traditional recruitment approaches. This research provides proof of concept that digital marketing may be an effective means of study recruitment for clinical research in PD and that it can be tailored to target specific PD cohort characteristics. Both highly targeted (late stage, early stage, geotargeting) and broad digital outreach not only attracted greater numbers of the PD subpopulation of interest, but also recruited more diverse demographic and clinical samples overall. Multi-pronged recruitment campaigns, such as Group 4’s broad online outreach, were the most effective with regards to both total number of new participants per 10-day period and sample heterogeneity.

Individuals historically underrepresented in PD research were recruited in higher numbers as a function of digital outreach than baseline recruitment practices. For example, digital strategies successfully attracted women, as well as participants who were older, non-white (African-American, Asian, American Indian, Multi-racial), Hispanic, lower income, less educated, and in worse physical health. This may be because traditional recruitment strategies oversample those with regular access to specialized PD care and time to participate in research. Social media also may be accessible and appealing to more diverse segments of the PD community than movement disorder specialty centers, or even traditional, in-person support groups.

With regards to ad visibility and cost, the average expenditure per new participant ranged from $21 to $108. Across campaigns, Facebook reached the greatest number of potential new participants for the lowest cost. Facebook also gave researchers the most control over ad optimization throughout the campaign, whereas many of the Google Ad refinements (e.g., keyword optimization, how and where ads were displayed among search results) were determined by internal Google algorithms and therefore inaccessible to the recruiting researchers. Overall, digital recruitment costs compared favorably to traditional approaches such as TV, radio, and newspaper announcements, as well as database mailings and flyering at PD community events.

### Applications to PD research at large

Although FI’s online cohort presents fewer barriers to participation than in-person clinical studies, several of the methods described herein may generalize across PD research settings. First, digital platforms and online interest-targeting strategies can enhance recruitment for both traditional and virtual PD studies, with methods selected based on budget and population characteristics of interest. In general, Facebook ads may be more cost-effective than Google ads. However, Google offers non-profit entities with appropriate charity status (e.g., philanthropic arms of educational institutions) small grants to cover the cost of a limited amount of digital advertising on their platform [35]. Second, broadly defined digital campaigns (based on one or two general criteria) may outperform narrowly defined campaigns (based on several specific criteria), in terms of overall recruitment numbers, as well as more diverse samples. Third, to help determine the utility of digital recruitment initiatives and monitor return on investment, researchers can use ad-specific URLs to track participant enrollment driven by unique online campaigns. Many digital platforms, such as Facebook, also provide ample opportunities to pilot test and optimize digital ads over time, based on their performance. Finally, effective recruitment should be multi-modal and employ various resources [5, 14, 15]. This proved critical even in FI, a virtual PD study not constrained by common recruitment barriers such as geographical location or level of health care access. For traditional PD studies, digital ads may provide an important supplement to clinic- and community-based recruitment.

This study is not without limitations. First, results are exploratory and reflect data from initial digital campaigns launched by FI. Second, data from this pilot study is cross-sectional and, as of yet, cannot speak to the longer-term retention and compliance of participants recruited via digital marketing vs. more traditional methods. That said, retention and compliance data for those enrolled in response all of these digital pilots will be tracked for the duration of the longitudinal cohort to inform unique retention initiatives for different cohorts of the PD population. Third, the FI platform is currently only available in English, which may limit participation in several regions of the world. Fourth, all individuals recruited via digital channels were at least moderate users of the internet; most either had active Facebook accounts and/or were actively researching PD via Google searches. Though research suggests that older adults are avid consumers of social media [27], it is important to note that online outreach methods may not generalize to those who are not computer literate and/or are too physically or cognitive impaired to effectively utilize the internet. Finally, as many of these recruitment campaigns were resource-intensive, methods may not generalize to trials of smaller size and scope.

In conclusion, these results offer preliminary evidence that targeted digital recruitment strategies may expand and diversify the recruitment pool for traditional PD studies, in addition to virtual trials. Given the marked increases in the size and diversity of the FI samples achieved via digital advertisements, future research will continue to explore other applications of precision-targeting and enrollment based on geography, socioeconomic status, and additional clinical characteristics, to better reflect the PD community in the demographic composition of research participants. These methods are anticipated to apply to both virtual and clinic-based PD research protocols worldwide, with the goal of improving representation in PD research and accelerating the advancement of clinical care.

## Supplementary Material

Supplementary Figures

## References

[1] Huang GD , Bull J , Johnston Mckee K , Mahon E , Harper B , Roberts JN (2018) Clinical trials recruitment planning: A proposed framework from the Clinical Trials Transformation Initiative. Contemp Clin Trials 66, 74–79. 29330082 10.1016/j.cct.2018.01.003

[2] Mathur S , DeWitte S , Robledo I , Isaacs T , Stamford J (2015) Rising to the challenges of clinical trial improvement in Parkinson’s disease. J Parkinsons Dis 5, 263–268. 25720445 10.3233/JPD-150541

[3] Carlisle B , Kimmelman J , Ramsay T , MacKinnon N (2015) Unsuccessful trial accrual and human subjects protections: An empirical analysis of recently closed trials. Clin Trials 12, 77–83. 25475878 10.1177/1740774514558307 PMC4516407

[4] (MJFF) MJFF, The Michael J. Fox Foundation Launches “Parkinson’s Clinical Trial Companion,” Educational Suite Designed to Support Progress in Parkinson’s Research by Increasing Patient Participation in Clinical Studies., https://www.michaeljfox.org/publication/michael-j-fox-foundation-launches-parkinsons-clinical-trial-companion-educational-suite.

[5] Berk S , Greco BL , Biglan K , Kopil CM , Holloway RG , Meunier C , Simuni T (2017) Increasing efficiency of recruitment in early Parkinson’s disease trials: A case study examination of the STEADY-PD III Trial. J Parkinsons Dis 7, 685–693. 29103052 10.3233/JPD-171199 PMC5676860

[6] Branson CO , Ferree A , Hohler AD , Saint-Hilarie M (2016) Racial disparities in Parkinson disease: A systematic review of the literature. Adv Parkinsons Dis 5, 87–96.

[7] Gilbert RM , Standaert DG (2020) Bridging the gaps: More inclusive research needed to fully understand Parkinson’s disease. Mov Disord 35, 231–234. 31710391 10.1002/mds.27906

[8] Schneider MG , Swearingen CJ , Shulman LM , Ye J , Baumgarten M , Tilley BC (2009) Minority enrollment in Parkinson’s disease clinical trials. Parkinsonism Relat Disord 15, 258–262. 18693062 10.1016/j.parkreldis.2008.06.005 PMC2700020

[9] Willis AW , Schootman M , Evanoff BA , Perlmutter JS , Racette BA (2011) Neurologist care in Parkinson disease: A utilization, outcomes, and survival study. Neurology 77, 851–857. 21832214 10.1212/WNL.0b013e31822c9123 PMC3162639

[10] Moore SF , Guzman NV , Mason SL , Williams-Gray CH , Barker RA (2014) Which patients with Parkinson’s disease participate in clinical trials? One centre’s experiences with a new cell based therapy trial (TRANSEURO). J Parkinsons Dis 4, 671–676. 25170676 10.3233/JPD-140432

[11] Tosserams A , Araújo R , Pringsheim T , Post B , Darweesh SKL , Inthout J , Bloem BR (2018) Underrepresentation of women in Parkinson’s disease trials. Mov Disord 33, 1825–1826. 30311268 10.1002/mds.27505

[12] Amit GS , Peter DRH , Akbar KW (2014) A primer on effectiveness and efficacy trials. Clin Transl Gastroenterol 5, e45. 24384867 10.1038/ctg.2013.13 PMC3912314

[13] Releases NIoHN, NIH announces national enrollment date for All of Us Research Program to advance precision medicine, https://www.nih.gov/news-events/news-releases/nih-announces-national-enrollment-date-all-us-research-program-advance-precision-medicine

[14] Sumedha C , Ashlie J , Ratna C , Caitlin RM , Ji Hyun K , Kayla Marie H , Yu-Ning W , Adele C , Diane KN , Knashawn HM , Ravishankar J (2018) Patient-centered recruitment and retention for a randomized controlled study. Trials 19, 1–10. 29587805 10.1186/s13063-018-2578-7 PMC5870194

[15] Picillo M , Kou N , Barone P , Fasano A (2015) Recruitment strategies and patient selection in clinical trials for Parkinson’s disease: Going viral and keeping science and ethics at the highest standards. Parkinsonism Relat Disord 21, 1041–1048. 26228079 10.1016/j.parkreldis.2015.07.018

[16] Smolensky L , Amondikar N , Crawford K , Neu S , Kopil CM , Daeschler M , Riley L , Brown E , Toga AW , Tanner C (2020) Fox Insight collects online, longitudinal patient-reported outcomes and genetic data on Parkinson’s disease. Sci Data 7, 67. 32094335 10.1038/s41597-020-0401-2 PMC7039948

[17] Rios Romenets S , Wolfson C , Galatas C , Pelletier A , Altman R , Wadup L , Postuma RB (2012) Validation of the non-motor symptoms questionnaire (NMS-Quest). Parkinsonism Relat Disord 18, 54–58. 21917501 10.1016/j.parkreldis.2011.08.013

[18] Goetz CG , Tilley BC , Shaftman SR , Stebbins GT , Fahn S , Martinez-Martin P , Poewe W , Sampaio C , Stern MB , Dodel R , Dubois B , Holloway R , Jankovic J , Kulisevsky J , Lang AE , Lees A , Leurgans S , Lewitt PA , Nyenhuis D , Olanow CW , Rascol O , Schrag A , Teresi JA , Van Hilten JJ , Lapelle N (2008) Movement Disorder Society-sponsored revision of the Unified Parkinson’s Disease Rating Scale (MDS-UPDRS): Scale presentation and clinimetric testing results. Mov Disord 23, 2129–2170. 19025984 10.1002/mds.22340

[19] D’Ath P , Katona P , Mullan E , Evans S , Katona C (1994) Screening, detection and management of depression in elderly primary care attenders. I: The acceptability and performance of the 15 item Geriatric Depression Scale (GDS15) and the development of short versions. Fam Pract 11, 260. 7843514 10.1093/fampra/11.3.260

[20] Jenkinson C , Fitzpatrick R , Peto V , Greenhall R , Hyman N (1997) The PDQ-8: Development and validation of a short-form parkinson’s disease questionnaire. Psychol Health 12, 805–814.

[21] Brennan L , Siderowf A , Rubright JD , Rick J , Dahodwala N , Duda JE , Hurtig H , Stern M , Xie SX , Rennert L , Karlawish J , Shea JA , Trojanowski JQ , Weintraub D (2016) The Penn Parkinson’s Daily Activities Questionnaire-15: Psychometric properties of a brief assessment of cognitive instrumental activities of daily living in Parkinson’s disease. Parkinsonism Relat Disord 25, 21–26. 26923524 10.1016/j.parkreldis.2016.02.020 PMC4818172

[22] Postuma RB , Arnulf I , Hogl B , Iranzo A , Miyamoto T , Dauvilliers Y , Oertel W , Ju YE , Puligheddu M , Jennum P , Pelletier A , Wolfson C , Leu-Semenescu S , Frauscher B , Miyamoto M , Cochen De Cock V , Unger MM , Stiasny-Kolster K , Livia Fantini M , Montplaisir JY (2012) A single-question screen for rapid eye movement sleep behavior disorder: A multicenter validation study. Mov Disord 27, 913–916. 22729987 10.1002/mds.25037 PMC4043389

[23] Chaudhuri KR , Sauerbier A , Rojo JM , Sethi K , Schapira AHV , Brown RG , Antonini A , Stocchi F , Odin P , Bhattacharya K , Tsuboi Y , Abe K , Rizos A , Rodriguez-Blazquez C , Martinez-Martin P (2015) The burden of non-motor symptoms in Parkinson’s disease using a self-completed non-motor questionnaire: A simple grading system. Parkinsonism Relat Disord 21, 287–291. 25616694 10.1016/j.parkreldis.2014.12.031

[24] Martínez-Martín P , Rodríguez-Blázquez C , Mario A , Arakaki T , Arillo VC , Chaná P , Fernández W , Garretto N , Martínez-Castrillo JC , Rodríguez-Violante M , Serrano-Dueñas M , Ballesteros D , Rojo-Abuin JM , Chaudhuri KR , Merello M (2015) Parkinson’s disease severity levels and MDS-Unified Parkinson’s Disease Rating Scale. Parkinsonism Relat Disord 21, 50–54. 25466406 10.1016/j.parkreldis.2014.10.026

[25] U.S. Census Bureau, 2010 Census Urban and Rural Classfication and Urban Area Criteria, https://www.census.gov/programs-surveys/geography/guidance/geo-areas/urban-rural/2010-urban-rural.html

[26] Giustini D , Ali SM , Fraser M , Boulos MNK (2018) Effective uses of social media in public health and medicine: A systematic review of systematic reviews. Online J Public Health Inform 10, e215. 30349633 10.5210/ojphi.v10i2.8270 PMC6194097

[27] Madden M, Older Adults and Social Media, https://www.pewresearch.org/internet/2010/08/27/older-adults-and-social-media/.

[28] Anderson M, Perrin A (2017, May) Tech adoption climbs among older adults. Pew Research Center. https://www.pewresearch.org/internet/2017/05/17/tech-adoption-climbs-among-older-adults/

[29] Clement J (2019, December) Distribution of Facebook users in the United States as of November 2019, by age group and gender. Statista. https://www.statista.com/statistics/187041/us-user-age-distribution-on-facebook/

[30] West C, Social media demographics to drive your brand’s online presence, https://sproutsocial.com/insights/new-social-media-demographics/#Facebook.

[31] Yandell K, Keeping Parkinson’s Disease a Secret, https://well.blogs.nytimes.com/2012/07/09/keeping-parkinsons-disease-a-secret/.

[32] ITV, Parkinson’s sufferers hiding ‘embarrassing’ symptoms, https://www.itv.com/news/2016-04-18/parkinsons-sufferers-hiding-embarrassing-symptoms/

[33] Fox S, Duggan M, Health Online 2013, https://www.pewresearch.org/internet/2013/01/15/health-online-2013/

[34] McCarrick K, In Case You Missed It: Michael J. Fox on “CBS Sunday Morning”, https://www.michaeljfox.org/news/case-you-missed-it-michael-j-fox-cbs-sunday-morning

[35] Google, Google Ad Grants, https://www.google.com/grants/eligibility/

