# Supplementary material for: Innovative Recruitment Strategies to Increase Diversity of Participation in Parkinson’s Disease Research: The Fox Insight Cohort Experience
Source: J Parkinsons Dis. 2020 Apr 3;10(2):665–75. doi: 10.3233/JPD-191901 (PMC7242847; doi:10.3233/JPD-191901)
Supplement: Supplementary Figures [file jpd-10-jpd191901-s001.pdf]

Supplementary Material

Innovative Recruitment Strategies to Increase Diversity of Participation in Parkinson's Disease Research: The Fox Insight Cohort Experience

Supplementary Figure 1. Sample Facebook Interest Targeting

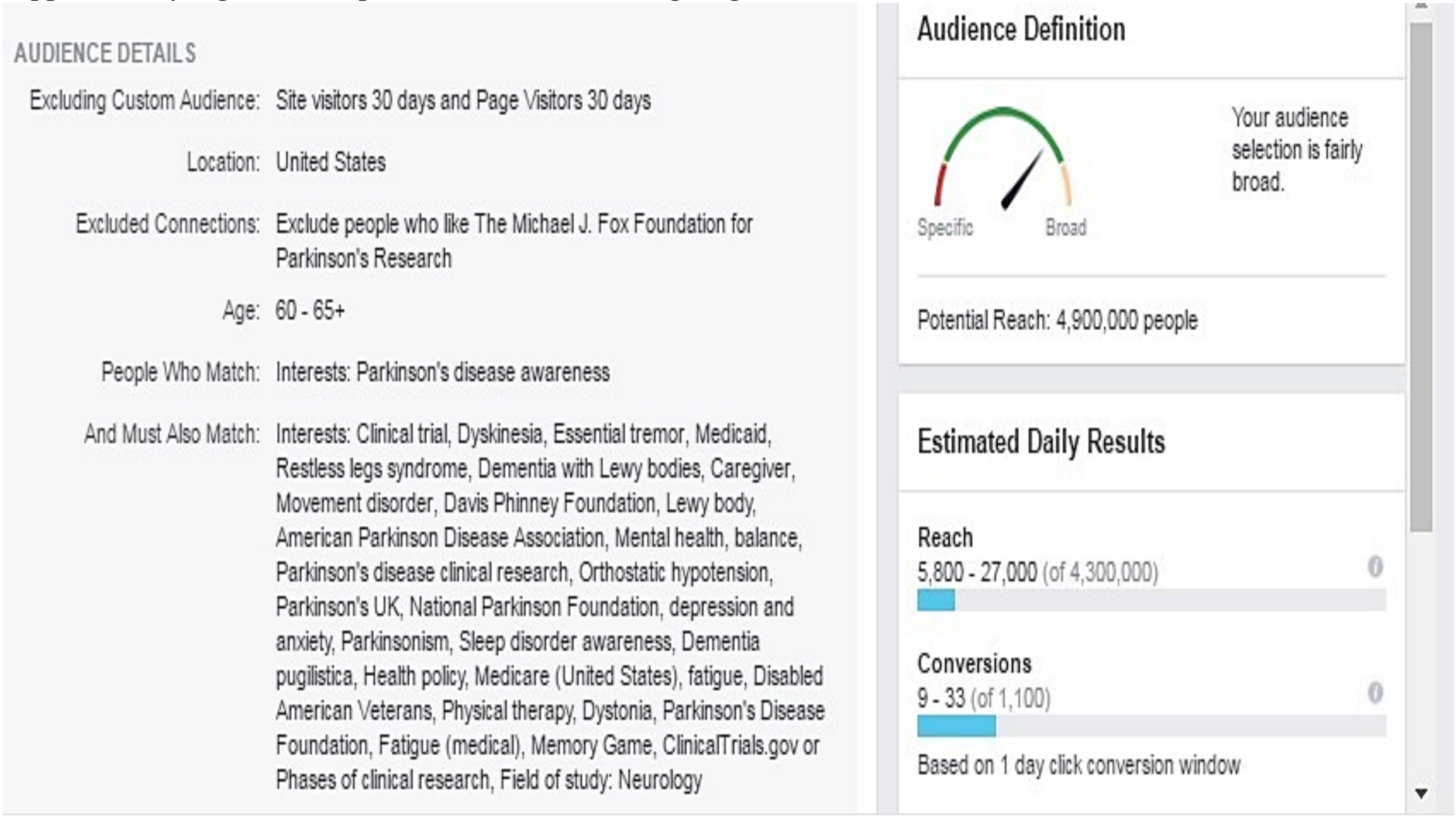

Supplementary Figure 2 (Panels 1-4). Sample Facebook Ads

1) FI Individual – Research Reimagined

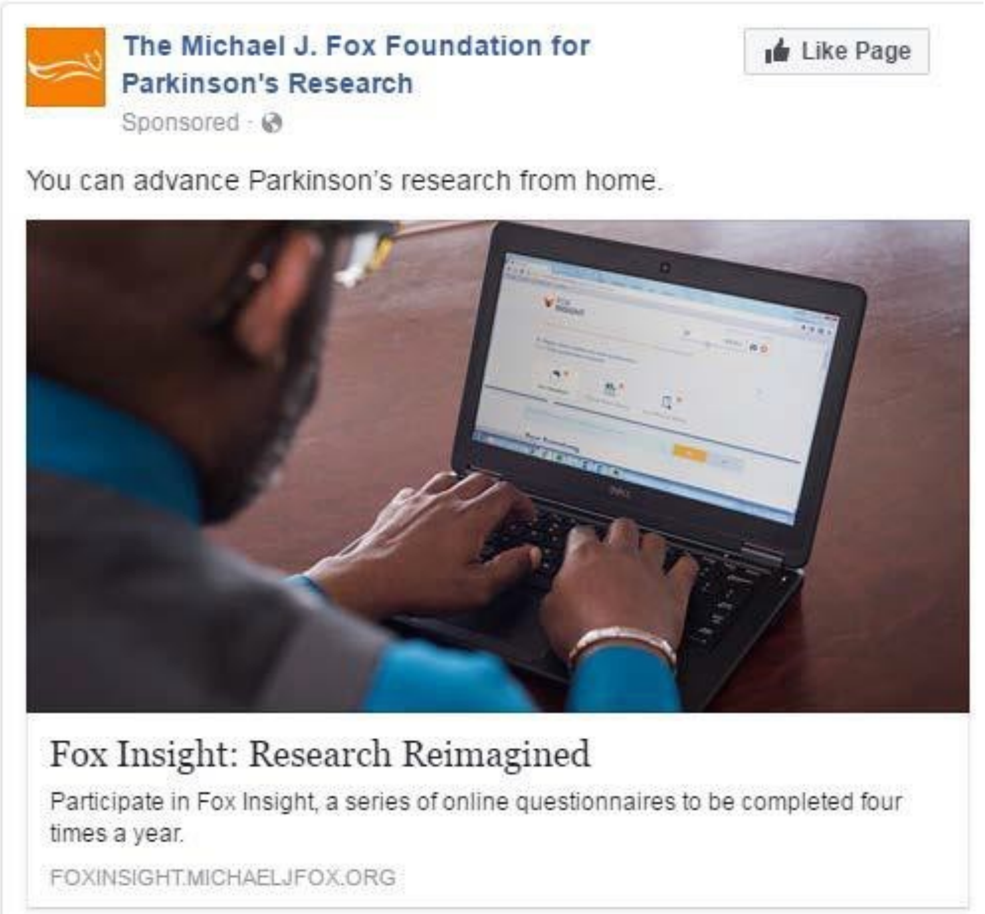

2) FI Individual – Generic

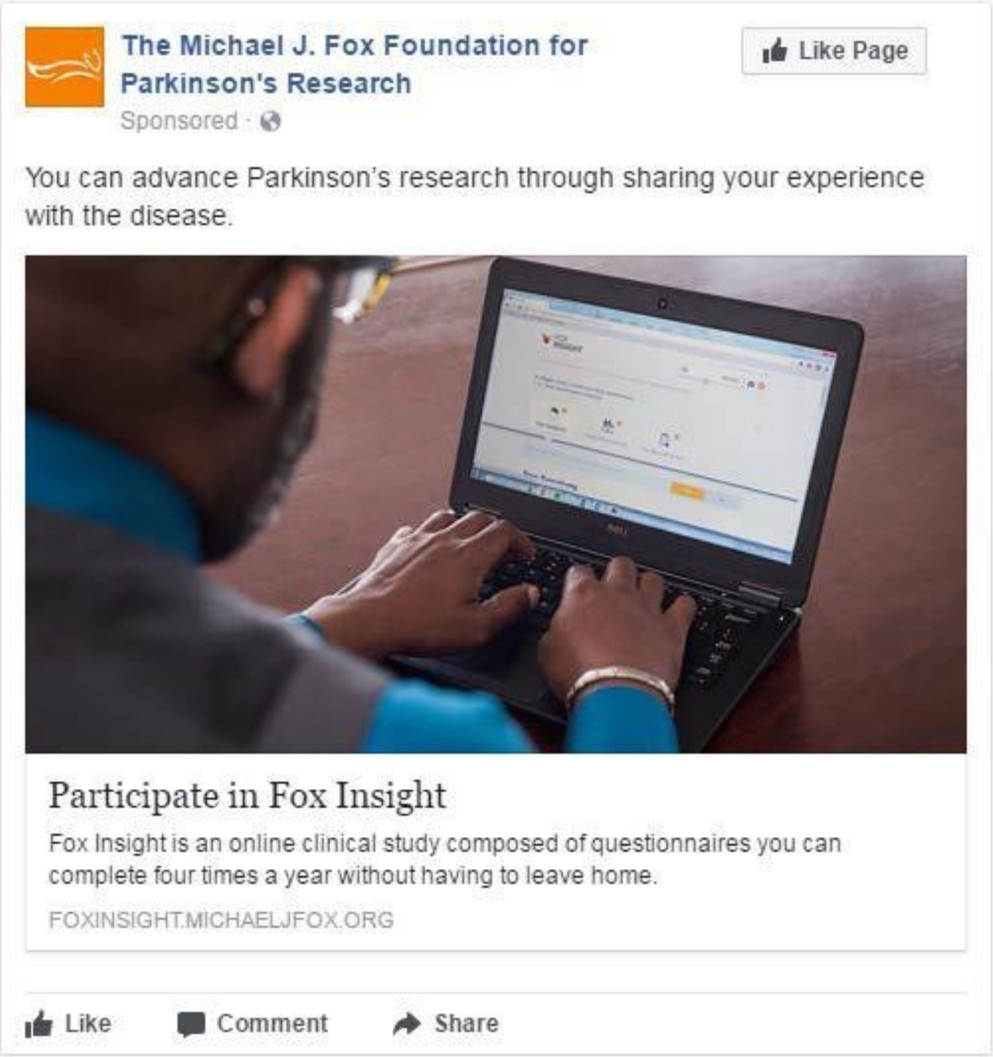

3) FI Collective – Impact the Future***

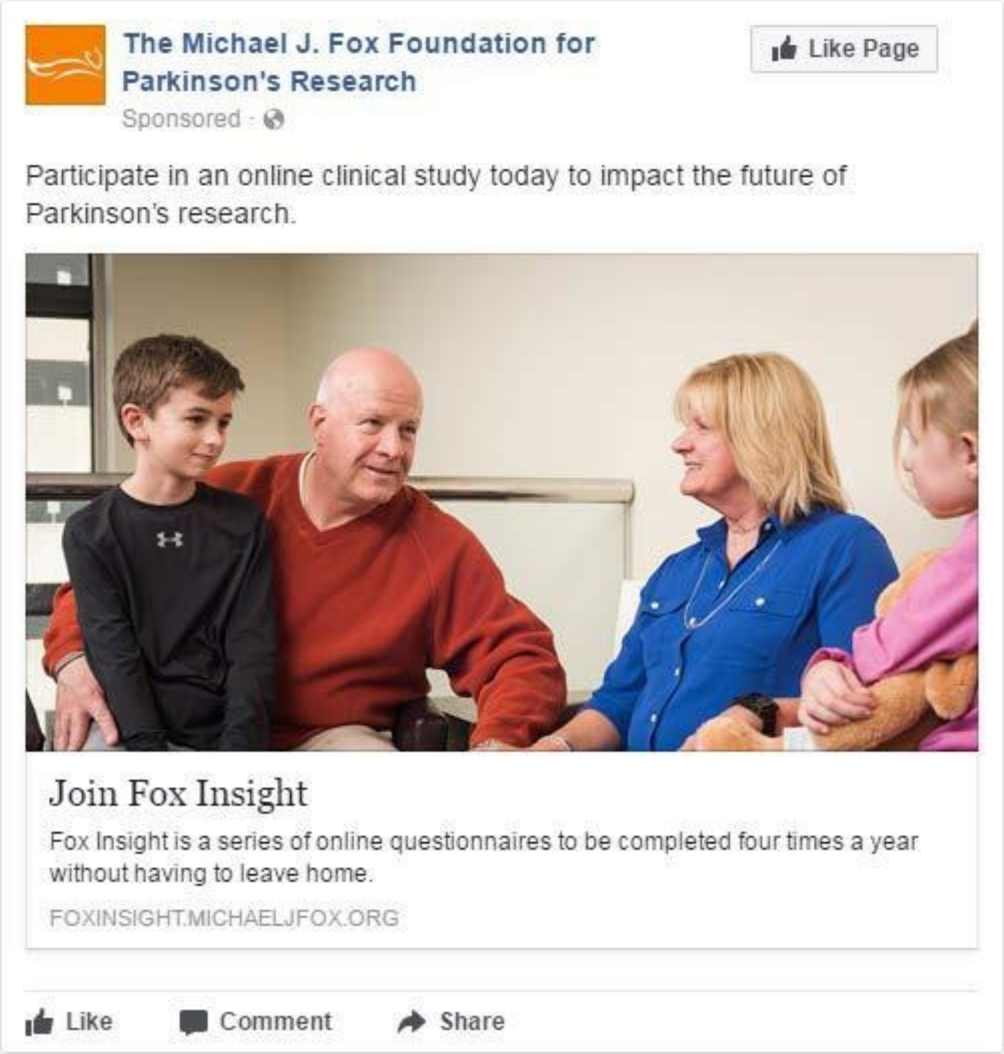

*****Winning Ad Creative**

4) FI Collective – Generic

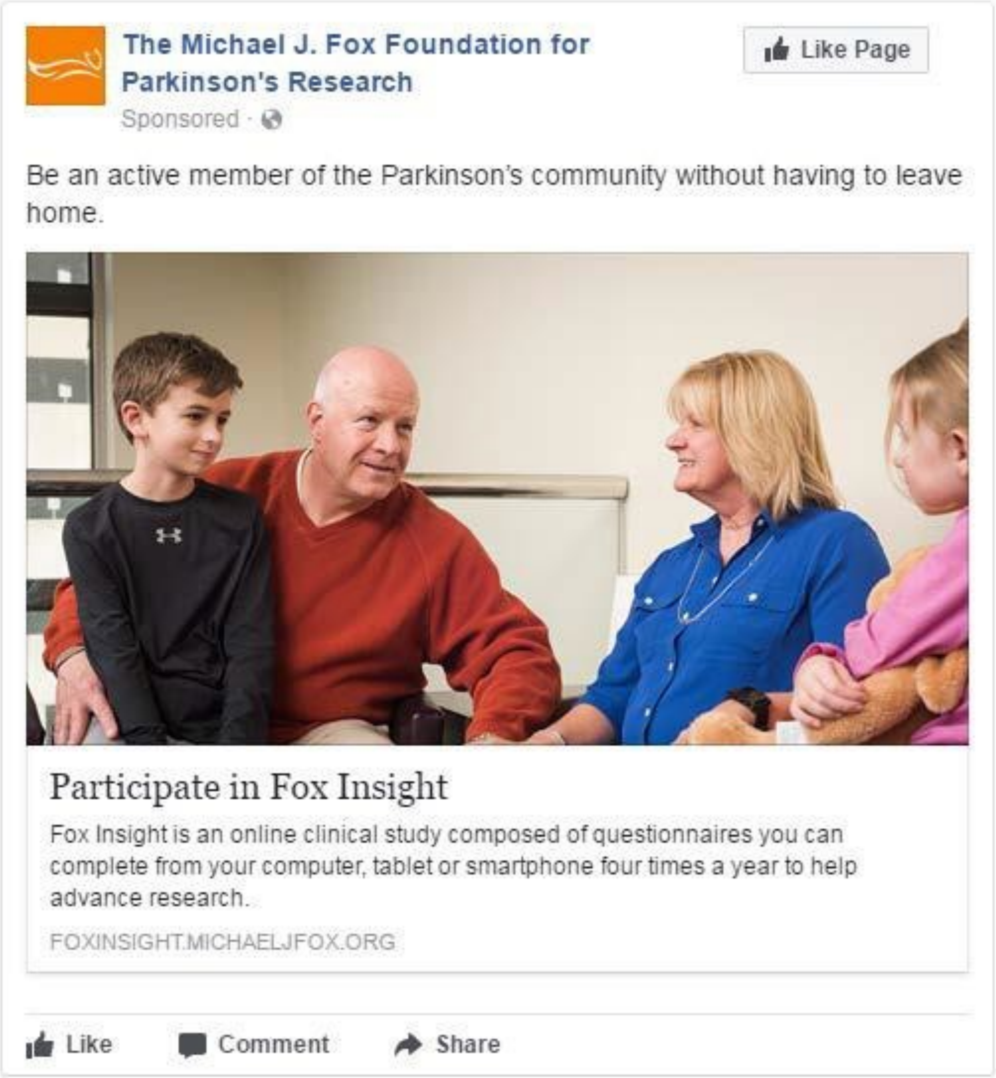

Supplementary Figure 3. Fox Insight Google AdWords

Final Keywords
Pre-diagnosis terms:
+parkinson’s tremor
+parkinson’s shaking
+parkinson’s symptoms
+parkinson’s definition
+parkinson’s doctor

Post-diagnosis terms:
+parkinson’s treatment
+parkinson’s drugs
+parkinson’s medication
+levodopa
+levodopa +side +effects
+deep +brain +stimulation
+parkinson's life expectancy
+parkinson’s prognosis
+parkinson’s stages
+parkinson’s support
+parkinson’s cause
+parkinson’s causes
+parkinson’s cure
+parkinson’s dopamine
+parkinson’s brain
+parkinson’s dyskinesia
+parkinson’s dystonia
+parkinson’s movement disorder
+parkinson’s movement disorder specialist

Late stage terms:
+parkinson’s assisted living +parkinson’s walker
+parkinson’s nursing home
+parkinson’s difficulty swallowing
+parkinson’s drooling
+parkinson’s dementia
+parkinson’s hallucinations
+parkinson’s psychosis
+parkinson’s memory
+parkinson’s falls

Supplementary Figure 4. Sample Google AD

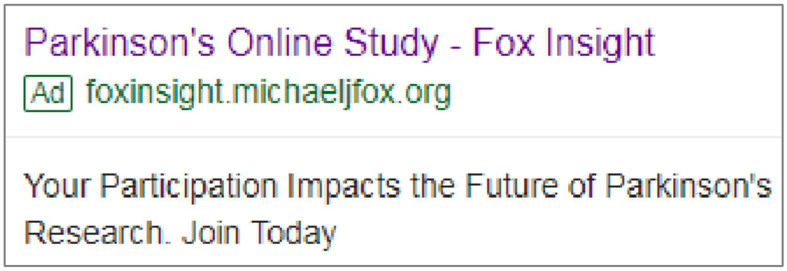

Supplementary Figure 5. Geotargeting States and Zip Codes

State	Targeted zip codes
Arizona; AZ	338
Idaho; ID	320
Kansas; KS	674
Montana; MT	404
North Dakota; ND	407
Nebraska; NE	535
New Mexico; NM	426
Nevada; NV	152
Oklahoma; OK	644
South Dakota; SD	385
Texas; TX	1969
Utah; UT	300
Wyoming; WY	195
